# Pepper Novel Serine-Threonine Kinase CaDIK1 Regulates Drought Tolerance *via* Modulating ABA Sensitivity

**DOI:** 10.3389/fpls.2020.01133

**Published:** 2020-07-23

**Authors:** Junsub Lim, Chae Woo Lim, Sung Chul Lee

**Affiliations:** Department of Life Science (BK21 Program), Chung-Ang University, Seoul, South Korea

**Keywords:** abscisic acid, drought stress, kinase, phosphorylation, stomata

## Abstract

Adaptation to drought stress is essential for plant growth and development. Plants often adapt to water deficit conditions by activating the ABA signaling. Here, we report that the pepper *CaDIK1* (*Capsicum annuum* Drought Induced Kinase 1) gene is essential for plant tolerance to drought stress. *CaDIK1* contains a serine-threonine kinase domain, which plays a role for attachment of phosphate to the target protein. The expression levels of *CaDIK1* are upregulated in pepper leaves by ABA, drought, NaCl and H_2_O_2_ treatments suggesting its role in abiotic stress response. We used *CaDIK1*-silenced pepper and *CaDIK1-*overexpressing (OX) transgenic Arabidopsis plants to evaluate their responses to ABA and drought. *CaDIK1-*silenced pepper plants conferred a reduced ABA sensitivity and drought hypersensitivity, which was accompanied by high levels of transpirational water loss. *CaDIK1-*OX plants displayed opposite phenotypes to *CaDIK1*-silenced peppers. In contrast, substitution of Lys350 to Asn in the kinase domain of CaDIK1 did not lead to alteration of drought sensitivity. Collectively, these data indicate that *CaDIK1* is a positive regulator of the ABA-mediated drought-stress tolerance.

## Introduction

As sessile organisms, plants face different environmental stresses, such as drought, high salinity, and cold. Drought stress is major component of these stresses, which restricts growth and development causing mortal damage like cell destruction to plants and limits agricultural productivity. To enhance drought tolerance, plants modulate their physiological and molecular states by regulating gene expression, post translational modifications, and stomatal closure (Lee and Luan, 2012). Plant response to drought stress has been well established, but the precise regulating mechanisms still remain unclear. Abscisic acid (ABA) is a critical plant hormone that regulates cellular processes, such as growth, development, and defense responses to abiotic stress (Lim et al., 2015a). When plants encounter drought stress, endogenous ABA is synthesized in several plant tissues and accumulated in leaves (Raghavendra et al., 2010) where it regulates the expression level of many stress response genes. Ultimately, the main response to increasing ABA levels is stomatal closure as it controls ion transport in guard cells (Geiger et al., 2009; Lee et al., 2009; Geiger et al., 2010). As a result, reduced transpirational water loss is enabling plants to tolerate drought stress.

Initiation of ABA signaling is from recognition of ABA by ABA receptors, thereby ABA signal is transduced to downstream target proteins *via* interaction with 2C type protein phosphatases (PP2Cs) (McCourt and Creelman, 2008; Gonzalez-Guzman et al., 2012). Protein phosphorylation and dephosphorylation play important roles in response to drought stress and in ABA signaling. SnRK2 type kinases and group A PP2Cs, core components of ABA signaling, are involved in drought response of plants (Lee and Luan, 2012; Lim et al., 2015a). Moreover, CBL-interacting protein kinases (CIPKs) and mitogen-activated protein kinases (MAPKs) positively and negatively function in drought stress and ABA signaling (Drerup et al., 2013; Ma et al., 2017; Cui et al., 2018). For instance, the SNF1-related protein kinase SnRK2.6/OST1 enhances drought tolerance by activating ABA binding factors (ABFs) and the slow anion channel-associated1 (SLAC1) (Yoshida et al., 2010; Yoshida et al., 2015).

Receptor-like kinases (RLKs), a family of transmembrane proteins, are also involved in the ABA signaling and, thus, in plants’ response to abiotic stress (Bai et al., 2009; Osakabe et al., 2010; Hua et al., 2012; Zhang et al., 2013; Lim et al., 2015b). They consist of an extracellular domain, a transmembrane domain, and an intracellular kinase domain (Shiu and Bleecker, 2001). Plant RLKs receive signals through their N-terminal extracellular domains (Shiu and Bleecker, 2003) and transfer these signals to their C-terminal intracellular domains, phosphorylating downstream factors to activate and amplify the signaling pathway (Gish and Clark, 2011). Molecular functions of leucine-rich repeat (LRR) containing RLKs in brassinosteroid signaling and pathogen recognition have revealed (Gomez-Gomez and Boller, 2000; Searle et al., 2003; Goff and Ramonell, 2007). Moreover, there are many RLKs are involved in abiotic stress and ABA signaling, including RPK1, PERK4, GHR1, CRK45, and LRK10L1.2 (Bai et al., 2009; Osakabe et al., 2010; Hua et al., 2012; Zhang et al., 2013; Lim et al., 2015b).

In this study, we present a novel RLK, *CaDIK1* (*Capsicum annuum* Drought Induced Kinase 1), isolated from pepper plants subjected to drought stress. *CaDIK1*-silenced pepper plants exhibited ABA hyposensitive and drought sensitive phenotypes. Contrarily, *CaDIK1* transgenic Arabidopsis plants showed enhanced ABA sensitivity and drought tolerant phenotypes. Moreover, we found that *CaDIK1* transgenic Arabidopsis plants expressed higher levels of ABA and drought response genes than did wild-type plants under drought conditions. Our findings imply that the CaDIK1 protein is involved in drought resistance by acting as a positive regulator of ABA signaling.

## Results

### Sequence Analysis of the Pepper *CaDIK1* Gene

**Figure 1:**
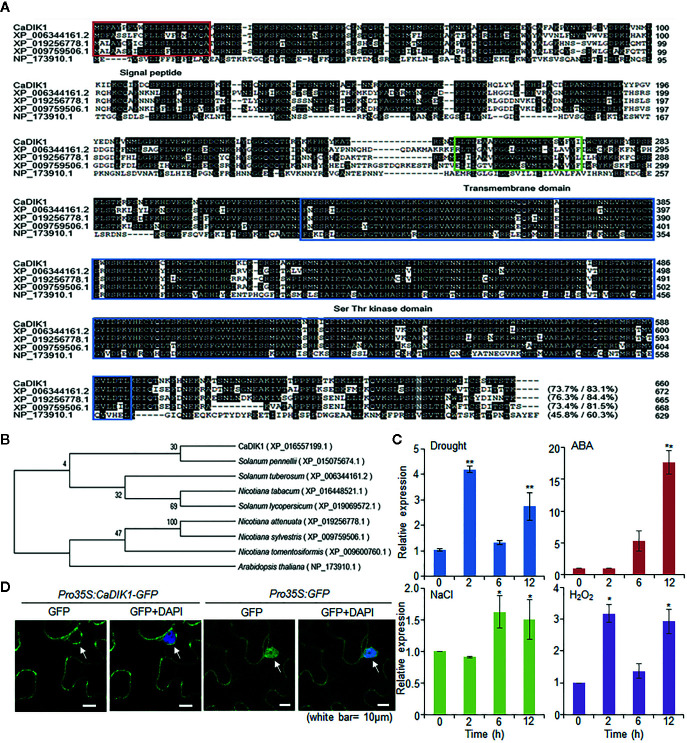
Molecular characterization of pepper CaDIK1 protein. **(A)** Alignment of CaDIK1 with its homologous proteins. Sequences include CaDIK1 (accession no. XP_016557199.1), *Solanum tuberosum* (accession no. XP_006344161.2), *Nicotiana attenuate* (accession no. XP_019256778.1), *Nicotiana sylvestris* (accession no. XP_009759506.1), and *Arabidopsis thaliana* (accession no. NP_173910.1). Identical and similar amino acid residues are highlighted in black. The red box indicates the signal peptide domain; the green box indicates the transmembrane domain; the blue box indicates the Ser-Thr kinase domain. **(B)** Phylogenetic tree analysis of CaDIK1 protein. BLAST search was performed using deduced amino acid sequences of *CaDIK1* gene, and sequences with highest similarity were gathered from each plant species. The phylogenetic tree was built and visualized using the neighbor-joining method with MEGA software (version 7.0). **(C)** Expression of *CaDIK1* in pepper leaves at various time points after treatment with drought, 100 μM abscisic acid (ABA), H_2_O_2_ (100 μm), and NaCl (200 mM). The pepper *Actin1* gene (*CaACT1*) was used as internal control. Data represent the mean ± standard error (SE) of three independent experiments; asterisks indicate significant difference compared with untreated control (0 h) (Student’s test; *P < 0.05, **P < 0.01). **(D)** Subcellular localization of the CaDIK1. The green fluorescent protein (GFP) fusion protein was used in *Nicotiana benthamiana* epidermal cells. 4′,6-Diamidino-2-phenylindole (DAPI) staining and white arrow mark the nucleus. White bar = 10 μm.

We isolated the pepper *CaDIK1* (*Capsicum annuum* Drought Induced Kinase 1 *)* gene from leaves of drought-treated pepper plants using the RNA-seq analysis (Lim and Lee, 2016). The *CaDIK1* cDNA contains 1983 bp and encodes a 660-amino acid residue with an isoelectric point of 6.3 and a measured molecular weight of 74.62 kD. Multiple sequence alignment analysis revealed a high amino acid sequence identity between CaDIK1 and other protein kinases (45.8–76.3%; **Figure 1A**). CaDIK1 contains a signal peptide, a transmembrane domain, and a C-terminal serine/threonine kinase domain. Its structure is similar to that of the AtLRK10L1.2 protein found in Arabidopsis and to the TaLRK10 protein found in wheat. AtLRK10L1.2 is involved in ABA signaling and drought tolerance, while TaLRK10 acts as a positive regulator of fungal resistance (Feuillet et al., 1997; Lim et al., 2015b). The phylogenetic tree reveals that CaDIK1 and its homologous protein sequences are phylogenetically distant (**Figure 1B**).

### *CaDIK1* Gene Induction and Subcellular Localization of CaDIK1

*CaDIK1* was isolated from drought-treated pepper leaves; hence we evaluated whether *CaDIK1* is induced by abiotic stress treatment. To determine the expression of *CaDIK1* under abiotic stress signaling, we performed qRT-PCR analysis using pepper leaves after treatments with drought, ABA, NaCl, and H_2_O_2_ (**Figure 1C**). Consistent with the RNA-seq results, drought treatment induced *CaDIK1* transcription in pepper leaves. Moreover, expression level of *CaDIK1* was induced by ABA, NaCl, and H_2_O_2_. These results suggest that *CaDIK1* is able to be involved in abiotic stress signaling.

Regarding the subcellular localization of the CaDIK1 protein, we observed a transient expression of the GFP-fused protein in the nuclear membrane and plasma membrane of leaf epidermal cells from *N. benthamiana* plants, which may be led by a transmembrane domain (from 248 to 270 aa) (**Figures 1A, D**). This indicates that CaDIK1 presumably functions in the cell membrane.

### Reduced Tolerance of *CaDIK1*-Silenced Pepper Plants to Drought Stress

**Figure 2:**
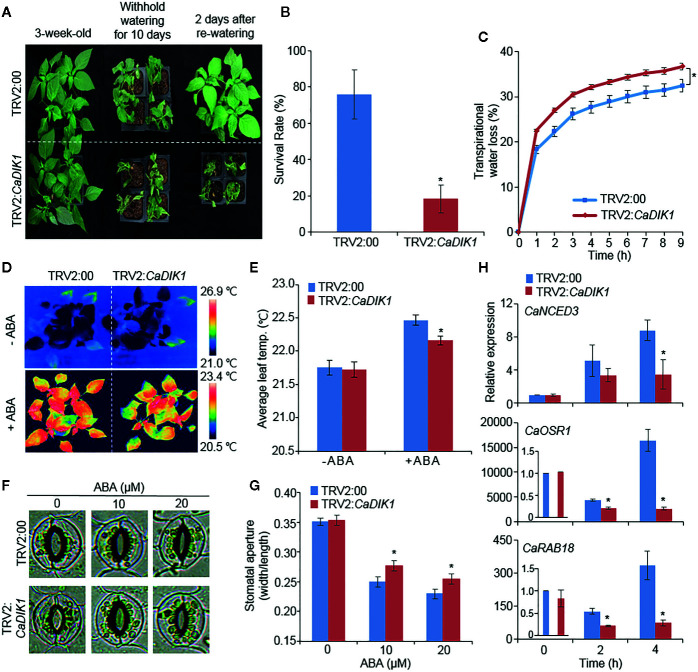
Reduced tolerance of *CaDIK1-*silenced pepper plants to drought stress. **(A)** Drought sensitive phenotype of *CaDIK1*-silenced pepper plants. Three-week-old TRV2:*CaDIK1* and TRV2:00 pepper plants were exposed to drought stress by withholding watering for 10 days and re-watering 2 days. Representative images were taken before (left) and after (middle) drought stress and re-watering (right). **(B)** Survival rates of plants after re-watering. Data represent the mean ± standard error of three biological replicates, each evaluating 20 plants. **(C)** Transpirational water loss from the leaves of empty vector control and *CaDIK1*-silenced pepper plants at various times after detachment of leaves. Data represent the mean ± standard deviation of three independent biological replicates. **(D**, **E)** Decreased leaf temperatures of *CaDIK1-*silenced pepper plants in response to ABA treatment. Leaf temperatures were measured 6 h after treatment with 100 μM ABA using thermal imaging and representative images were taken. **(D)**; the mean leaf temperature was measured using 12 plants of each line **(E)**. Data represent the mean ± standard deviation of three independent experiments. Data represent the mean ± SD of three independent biological replicates. **(F**, **G)** Stomatal apertures in empty vector control and *CaDIK1*-silenced pepper plants after ABA treatment. Representative images were taken under a microscope **(F)** and the stomatal apertures were measured **(G)**. Leaf peels were harvested from 2-week-old pepper plants and incubated in stomatal opening solution containing 0, 10, and 20 μM ABA; the stomatal apertures were then measured under a microscope. Representative images were taken at 2.5 h after various concentrations of ABA treatment. Data represent the mean ± standard error of three independent experiments, each evaluating 20 plants. **(H)** Transcript expression of drought-inducible genes using quantitative reverse transcription polymerase chain reaction in control and *CaDIK1*-silenced pepper plants exposed to drought stress at 0, 2, and 4 h after detachment. The relative expression levels (δδCT) of each gene were normalized to the geometric mean of *CaACT1* as an internal control gene. Data represent the mean ± standard error of three independent biological replicates, each evaluating 20 plants. Asterisks indicate significant differences between the control and the *CaDIK1*-silenced pepper plants (Student’s *t*-test; *P < 0.05).

To investigate the role of *CaDIK1* in drought response, we performed a virus-induced gene silencing (VIGS) analysis (Lim and Lee, 2014). To confirm the efficiency of VIGS, we conducted a RT-PCR analysis, which revealed that the expression levels of *CaDIK1* was lower in *CaDIK1*-silenced pepper plants (TRV2:*CaDIK1*) than in control plants (TRV2:00) (**Supplementary Figure 1A**). Under normal growth conditions, no differences were observed between *CaDIK1*-silenced and control pepper plants (**Figure 2A**; left panel). However, after being subjected to the drought treatment and rewatering, *CaDIK1*-silenced pepper plants showed wilted phenotypes in comparison to control plants (**Figure 2A**; middle and right panels). Additionally, the survival rate of control plants was higher (76%) in comparison to *CaDIK1*-silenced pepper plants (18.3%; **Figure 2B**). To determine whether the drought-sensitive phenotype of the *CaDIK1*-silenced pepper plants was attributable to difference of water retention capacity, we measured the fresh weight of detached first and second leaves (**Figure 2C**). The transpirational water loss was higher in *CaDIK1*-silenced pepper leaves than the control leaves. Previous studies have suggested that the transpiration rate is controlled by ABA sensitivity (Lim et al., 2017; Baek et al., 2018). To determine the ABA sensitivity, the leaf temperatures and stomatal apertures were measured with or without treatment of ABA (**Figures 2D–G**). In the absence of ABA, there were no significant differences between the *CaDIK1*-silenced pepper and control plants. Even though leaves treated with ABA showed higher temperatures and reduced stomatal aperture in both plant types, *CaDIK1*-silenced pepper plants exhibited lower leaf temperatures (**Figures 2D, E**) and larger stomatal pores (**Figures 2F, G**) in comparison to control plants.

Previous studies suggested that altered responses to drought stress are related with the expression level of stress-related genes (Lim et al., 2017; Lim et al., 2018) and *CaDIK1* expression is positively correlated with drought tolerance and ABA sensitivity; hence, we investigated whether enhanced expression of *CaDIK1* affects the expression levels of stress-related genes (**Figure 2H**). To examine this, qRT-PCR analysis was performed with stress-related genes in drought stress treated pepper leaves. The expression levels of these genes, including *CaNCED3*, *CaOSR1*, and *CaRAB18* were less induced in *CaDIK1-*silenced plants than in control pepper plants. Since *CaNCED3* is associated with ABA biosynthesis (Lim et al., 2017; Lim et al., 2018), we measured ABA content in control plants and *CaDIK1*-silenced plants after drought treatment. Consistent with the expression level of *CaNCED3* gene, *CaDIK1*-silenced plants displayed low ABA content compared to WT (**Supplementary Figure 2A**). These results indicate that the differences observed in *CaDIK1*-silenced pepper plants that were subjected to drought stress were caused by suppressing the expression of the *CaDIK1.*

### Enhanced Sensitivity of *CaDIK1*-OX Plants to ABA

**Figure 3:**
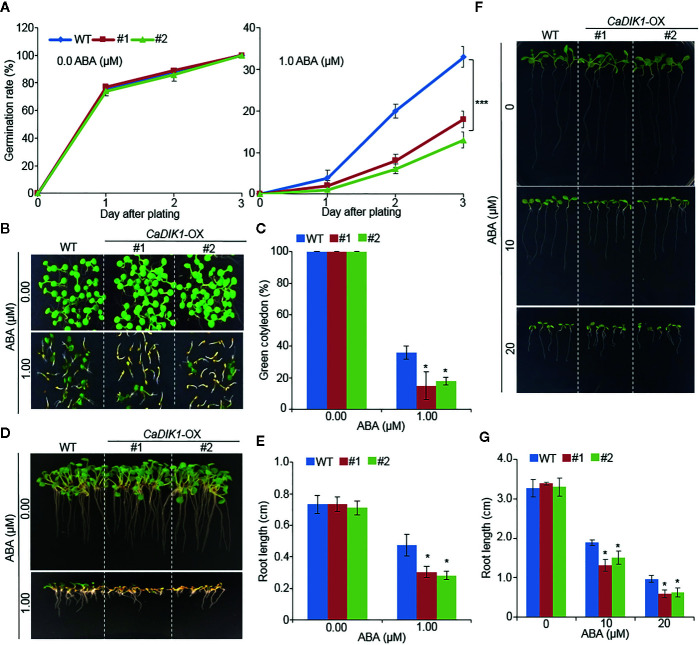
Enhanced ABA sensitivity of *CaDIK1-*OX plants. **(A)** Seed germination rate of *CaDIK1*-OX mutants and wild-type (WT) plants on 0.5× MS medium containing 1.0 μM of ABA. Germination rate was measured 0–3 days after plating. Data represent the mean ± standard error of three independent biological replicates, each evaluating 100 seeds. **(B)** ABA-mediated inhibition of seedling development of *CaDIK1*-OX mutants and wild-type plants with 0.0, 1.0 μM of ABA. Representative images were taken 10 days after plating. **(C)** The number of seedlings in each line with expanded green cotyledons was quantified 10 days after plating. Data represent the mean ± standard error of three independent biological replicates, each evaluating 25 seeds. **(D**, **E)** Primary root elongation of wild-type and transgenic lines in response to ABA. Representative images were taken **(D)** and root length of each plant was measured 7 days after plating **(E)**. Data represent the mean ± standard error of four independent biological replicates, each evaluating 25 seeds. Different letters indicate significant differences between wild-type and transgenic lines (Student’s *t*-test; *P* < 0.05). **(F**, **G)** Primary root elongation of wild-type and transgenic plants exposed to ABA after germination. Three-day-old seedlings grown on 0.5× MS medium were transferred to fresh 0.5× MS medium containing 0 µM or 10 µM, 20 µM ABA. After 7 days, the representative images were taken **(F)**, and the root length in each line was measured **(G)**. Data represent the mean ± standard error of three independent biological replicates, each evaluating 50 seeds. Asterisks indicate significant differences between wild-type and transgenic plants (Student’s *t*-test; *P* < 0.05).

To investigate further biological function of *CaDIK1*, we generated Arabidopsis transgenic plants that overexpressed *CaDIK1* and obtained two independent T_3_ homozygous transgenic lines (*CaDIK1-*OX #1 and #2) that showed a high expression of *CaDIK1* (**Supplementary Figure 1B**). Without ABA, germination rates of wild-type and *CaDIK1*-OX seeds were not significantly different. However, in the presence of ABA, germination rates of *CaDIK1*-OX seeds were significantly lower than those of wild-type seeds (**Figure 3A**). We analyzed seedling establishment and primary root growth in response to ABA (**Figures 3B–E**). Differences between both plants were also distinguishable in the seedling stages. At 10 days after plating, the rate of cotyledon greening was lower in *CaDIK1*-OX plants than in the wild-type plants (**Figures 3B, C**). Consistent with seedling establishment, primary root growth of the *CaDIK1*-OX plants was severely impaired by ABA treatment as compared with wild-type plants (**Figures 3D, E**). To examine whether the ABA sensitivity in cotyledon greening and primary root growth is caused by the post-germination growth, 3-days-old seedling germinated on MS media without ABA were transferred to ABA treated media. In consistent with germination growth, post-germination growth in *CaDIK1*-OX plants also showed ABA sensitive phenotypes compared with wild-type plants (**Figures 3F, G**). These results suggest that enhanced expression of the *CaDIK1* confers hypersensitivity to ABA in germination and post-germination growth.

### Enhanced Tolerance of *CaDIK1*-OX Plants to Drought Stress

**Figure 4:**
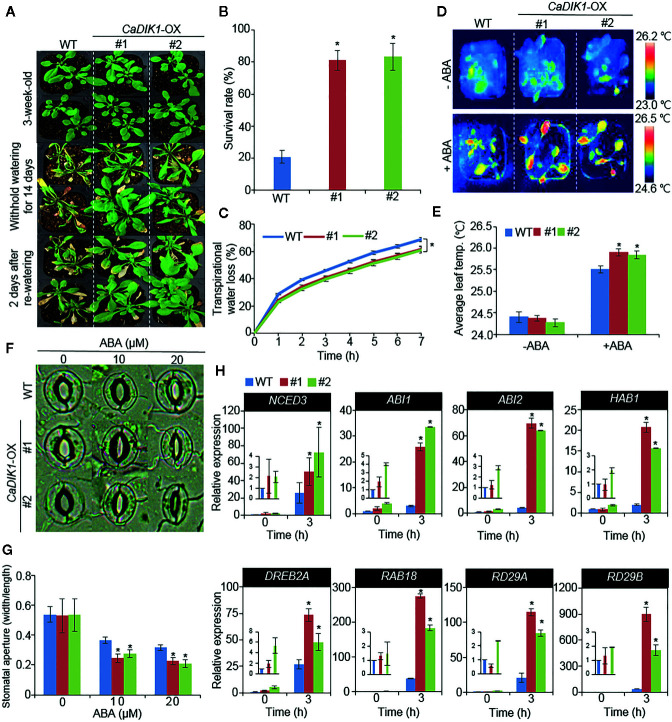
Enhanced drought tolerance of *CaDIK1-*OX plants. **(A)** Drought stress phenotype of wild-type (WT) and *CaDIK1-*OX transgenic plants. Three-week-old wild-type and transgenic plants were subjected to drought stress by withholding watering for 14 days and re-watering for 2 days. **(B)** Survival rates of plants after re-watering. Data represent the mean ± SE of three independent biological replicates, each evaluating 30 plants. **(C)** Transpirational water loss of detached rosette leaves in wild-type and transgenic plants leaves at various time points. Data represent the mean ± standard error of three independent biological replicates, each evaluating 50 leaves. **(D**, **E)** Enhanced leaf temperatures of *CaDIK1*-OX plants in response to abscisic acid (ABA) treatment. Representative thermographic images of wild-type and *CaDIK1-*OX plants 6 h after treatment with 100 μm ABA (D); the mean leaf temperatures of the three largest leaves were measured using 10 plants of each line **(E)**. Data represent the mean ± standard deviation of three independent biological replicates, each evaluating 10 plants. **(F**, **G)** Stomatal apertures in wild-type and *CaDIK1-*OX plants treated with ABA. Leaf peels were harvested from three-week-old plants of each line and incubated in stomatal opening solution containing 0, 10, or 20 μM ABA. Representative images were taken under a microscope **(F)** and stomatal apertures were measured **(G)**. Data represent the mean ± standard error of three independent biological replicates. **(H)** Transcript expression of drought-inducible genes using quantitative reverse transcription polymerase chain reaction in *CaDIK1*-OX plants exposed to drought stress at 3 h after detachment. The relative expression levels (δδCT) of each gene were normalized to the geometric mean of *Actin8* as an internal control gene. Data represent the mean ± standard error of three independent biological replicates. Asterisks indicate significant differences between wild-type and transgenic lines (Student’s *t*-test; *P < 0.05).

To verify if the enhanced expression of *CaDIK1* alters drought response, both wild-type and *CaDIK1*-OX plants were subjected to drought stress (**Figure 4**). Under normal growth conditions, both plants showed unaltered phenotypes (**Figure 4A**, upper panel). However, after withholding water and rewatering, wild-type plants exhibited a more wilted phenotype than did *CaDIK1*-OX plants (**Figure 4A**, middle and under panels). Moreover, only 20% of wild-type plants resumed growth, whereas approximately 80% of *CaDIK1*-OX plants resumed growth and survived (**Figure 4B**). Consistently, the relative water contents of *CaDIK1*-OX plants were higher than those of WT plants after treatment with drought stress (**Supplementary Figure 3**) and transpirational water loss in *CaDIK1*-OX plants was lower than that in wild-type plants (**Figure 4C**), indicating that the enhanced tolerance to drought stress could be attributed to altered water retention capacity. Generally, drought sensitivity and tolerance in plants are determined by at least two or three cellular and molecular parameters. Previous studies monitored ABA sensitivity, which determines drought sensitivity and tolerance, using measuring the leaf temperatures and the stomatal aperture (Lim et al., 2017; Baek et al., 2018). Moreover, expression level of stress-related genes affects the plants response to drought stress. Hence, we measured the leaf temperatures, stomatal apertures, and stress related gene expression (**Figures 4C–H**). In the absence of ABA, leaf temperatures and stomatal apertures were not significantly different in both plants. However, in comparison to wild-type plants, *CaDIK1*-OX plants showed that higher leaf temperatures and reduced stomatal apertures (**Figures 4D–G**).

Next, we investigated whether enhanced expression of *CaDIK1* affects the expression levels of stress-related genes (**Figure 4H**). To test this, qRT-PCR analysis was performed with stress-related genes in drought stress untreated or treated leaves. The expression levels of these genes, including *NCED3*, *DREB2A*, *RAB18*, *RD29A*, and *RD29B*, were more induced in *CaDIK1-*OX plants than in wild-type plants. Also, ABA content was consistently higher in *CaDIK1-*OX plants on drought treatment (**Supplementary Figure 2B**). The enhanced expressions of stress-related genes may affect the drought tolerance of *CaDIK1-*OX plants.

### *CaDIK1*-OX Regulates Drought Response Through Kinase Domain

**Figure 5:**
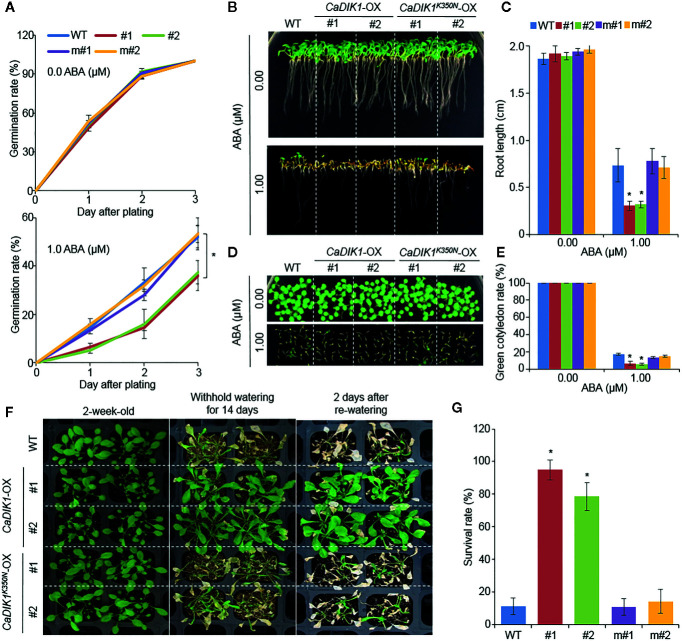
Phenotypic analysis of *CaDIK1^K350N^-*OX transgenic *Arabidopsis* lines under ABA and Drought stress. **(A)** Seed germination rate of and wild-type (WT), *CaDIK1-*OX, and *CaDIK1^K350N^-*OX plants on 0.5× MS medium containing 0.0 and 1.0 μM of ABA. Germination rate was measured 0–3 days after plating. Data represent the mean ± standard error of three independent biological replicates, each evaluating 100 seeds**. (B**, **C)** Primary root elongation of wild-type and transgenic lines in response to ABA. Representative images were taken **(B)** and root length of each plant was measured 7 days after plating **(C)**. Data represent the mean ± standard error of three independent biological replicates, each evaluating 30 seeds. **(D**, **E)** ABA-mediated inhibition of seedling development of *CaDIK1^K350N^-*OX mutants and wild-type plants with 0.0 and 1.0 μM of ABA. Representative images were taken **(D)** and the number of seedlings in each line with expanded green cotyledons was quantified 7 days after plating **(E)**. Data represent the mean ± standard error of three independent biological replicates, each evaluating 25 seeds. **(F)** Drought stress phenotype of *CaDIK1^K350N^-*OX transgenic plants. Two-week-old wild-type and transgenic plants were subjected to drought stress by withholding watering for 14 days and re-watering for 2 days. **(G)** Survival rates of plants after re-watering. Data represent the mean ± standard error of three independent biological replicates, each evaluating 30 plants. Asterisks indicate significant differences between wild-type and transgenic lines (Student’s *t*-test; *P < 0.05). m#1: *CaDIK1 ^K350N^-*OX *#1*; m#2: *CaDIK1 ^K350N^-*OX *#2*.

To investigate how the kinase domain of CaDIK1 is important in ABA and drought responses, we mutated the CaDIK1 kinase domain (350 lysine to asparagine: K350N) generating *CaDIK1^K350N^*–OX transgenic Arabidopsis plants (**Figure 5**). We selected two independent lines and performed phenotype assays with wild-type and *CaDIK1*-OX plants. In normal growth conditions, *CaDIK1^K350N^*–OX plants showed no phenotypic differences in comparison to wild-type and *CaDIK1*-OX plants. However, when plants were exposed to ABA, wild-type and *CaDIK1^K350N^*-OX plants showed higher germination rates, root lengths, and percentage of green cotyledons in comparison to *CaDIK1*-OX plants (**Figures 5A–E**). To examine drought stress response, these plants were subjected to drought treatment that consisted of withholding water for 14 days followed by re-watering for 2 days (**Figure 5F**). Wild-type and *CaDIK1 ^K350N^*-OX plants exhibited wilted phenotypes relative to *CaDIK1*-OX plants (**Figure 5F**: middle and right panels). *CaDIK1^K350N^*-OX and wild-type plants showed lower survival rates in comparison to *CaDIK1*-OX plants (**Figure 5G**). Moreover the relative MDA and proline contents of *CaDIK1*-OX plants were lower and higher, respectively, than those of wild-type plants after dehydration treatment (**Supplementary Figure 4**). These data indicate that the kinase domain of CaDIK1 is essential for ABA and drought responses.

## Discussion

Plants are affected by water-deficit conditions, including drought, high salt, and extreme temperatures. Plant kinases are involved in plant stress responses, including drought stress. Among them, RLKs are the most well studied kinase proteins related to stress response in plants (Shiu and Bleecker, 2003). They are anchored on the plasma membrane and consist of an external N-terminal region, a transmembrane domain, and an internal C-terminal region (Shiu and Bleecker, 2003). Under drought stress conditions caused by water deficit, RLKs of many plants sense a stress signal through their external N-terminal region, transporting and amplifying that signal downstream *via* phosphorylation to target protein through their internal C-terminal kinase domain (Shiu and Bleecker, 2003; Gish and Clark, 2011). For example, a brassinosteroid signaling component, BRI1-Associated Receptor Kinase 1 (BAK1), regulates ABA-mediated stomatal closure by phosphorylating OST1 (Shang et al., 2016). Another RLK, Guard Cell Hydrogen Peroxide-Resistant1 (GHR1), regulates stomatal closure by phosphorylating and activating the SLAC1 channel (Hua et al., 2012). Moreover, previous studies have shown that AtLRK10L1.2 is involved in ABA signaling and positively regulates drought resistance (Lim et al., 2015b). In this study, we identified a drought-induced RLK in pepper plants, CaDIK1, which acts as a positive regulator of drought stress response *via* ABA-mediated signaling.

Expression levels of *CaDIK1* were induced by drought, high salinity conditions, and presence of ABA. Owing to the low transformation efficiency and technical restriction involved in genetic investigation in pepper plans, we used VIGS to generate *CaDIK1-*silenced pepper plants (loss of function) and *CaDIK1-*OX in Arabidopsis plants (gain-of function). *CaDIK1-*silenced pepper plants exhibited severely wilted phenotypes and lower survival rates in drought condition. Drought resistance is acquired through several factors, such as stomatal closure, anthocyanin accumulation, and reactive oxygen species (ROS)-scavenging enzymes (Varshney et al., 2018). Stomatal closure is a major short-term factor of drought response that is related to ABA signaling (Lim et al., 2015a). When plants close their stomata, transpirational water loss is reduced and leaf temperatures are increased in comparison to conditions when stomata are open. Detached leaves of *CaDIK1*-silenced pepper plants showed higher transpirational water loss and lower leaf temperatures than did control plants, indicating that lower expression levels of *CaDIK1* lead to reduced ability of stomatal closure. Contrarily, *CaDIK1-*OX plants showed increased ABA sensitivity in seeds and seedlings, and drought tolerant mature plants. Moreover, *CaDIK1^K350N^*-OX plants showed phenotypes similar to those of wild-type plants under treatments with ABA and drought stress. These results indicate that *CaDIK1* positively regulates drought stress response through ABA-mediated signaling as CaDIK1 acquires kinase domain.

Many stress responsive genes, related to ABA biosynthesis, ABA signaling, and stress defense response, are induced when plants face different stress conditions. For instance, *NCED3* plays a key role in ABA synthesis and group A PP2Cs are core ABA signaling components, acting as negative regulators in the ABA signaling pathway (Lim et al., 2015a; Lim and Lee, 2016; Baek et al., 2017). In particular, the expression levels of these PP2C genes increase under drought stress through the negative feedback regulatory loop (Merlot et al., 2001). Our qRT-PCR data showed that stress response genes have higher expression levels in *CaDIK1*-OX plants than in wild-type plants. Consistent with the expression level, *CaDIK1*-OX plants have higher ABA content than wild-type plants (**Supplementary Figure 2**). Additionally, the expression of stress responsive genes involved in ABA-dependent and independent pathways, such as *RAB18*, *RD29B*, *RD28A*, and *DREB2A*, was regulated by CaDIK1. These data indicate that CaDIK1 may act upstream in relation to *NCED3* and group A PP2Cs and also ABA biosynthesis and all processes involved in the ABA signaling pathway are important in drought tolerant *CaDIK1*-OX plants. However, it remains unclear.

In conclusion, we suggest that CaDIK1 positively regulates the ABA signaling and drought stress response. The kinase domain of CaDIK1 regulates ABA signaling by controlling stomatal closure and drought-responsive gene expression. However, in this study it still remains unclear how CaDIK1 modules ABA biosynthesis and ABA signaling directly or indirectly and which downstream genes are regulated by the kinase domain of CaDIK1. Therefore, further studies should focus on identifying downstream elements that interact with and are phosphorylated by CaDIK1.

## Materials and Methods

### Plant Materials

We used pepper (*C. annuum* L., cv. Nockwang), tobacco (*Nicotiana benthamiana*), and *Arabidopsis thaliana* (ecotype Col-0) plants. Seeds of all species were sown in a steam-sterilized compost mix of soil (peat moss, perlite, and vermiculite, 5:3:2, v/v/v), sand, and loam (1:1:1, v/v/v). Plants were placed in growth chambers at 24 ± 1°C under white fluorescent light (130 μmol photons·m^-2^·s^-1^) with a 16 h light/8 h dark cycle. Arabidopsis seeds were treated at 4°C for 2 days before being placed in the growth chambers.

### Virus-Induced Gene Silencing

Virus-induced gene silencing (VIGS) was used with the tobacco rattle virus (TRV) to generate knock-down *CaDIK1* in pepper plants according to Lim et al. (2015b). A 300-bp fragment (136-435) of the *CaDIK1* cDNA was inserted into the pTRV2 vector. A GV3101 strain of *Agrobacterium tumefaciens* containing pTRV1, pTRV2:00, and pTRV2:*CaDIK1* was co-infiltrated into fully expanded cotyledons of pepper plants (OD600 = 0.2 for each construct).

### Transgenic Arabidopsis Plants

To generate *CaDIK1*-overexpressing (OX) Arabidopsis transgenic plants, we used the p326GFP binary vector containing coding regions for *CaDIK1* without the stop codon. A GV3101 strain of *A.* *tumefaciens* containing the 35S:*CaDIK1*- GFP construct was inoculated into Arabidopsis using the floral dip method (Clough and Bent, 1998). All transgenic plants were generated in the Col-0 background. To select *CaDIK1*-OX plants, seeds were harvested from the transgenic plants and sown on MS medium containing 25 μg·ml^-1^ phosphinothricin.

### ABA, Drought, NaCl, and H_2_O_2_ Treatments

To analyze *CaDIK1* expression patterns in pepper plants, six-leaf stage pepper plants were treated with ABA (100 μM), H_2_O_2 (_100 μM), drought, or NaCl (200 mM) as described previously (Lim et al., 2015b). Pepper leaves were harvested at different times after treatments were carried out and were subjected to quantitative real-time transcription-polymerase chain reaction (qRT-PCR) analysis. The qRT-PCR analysis was also performed in 4-week-old *CaDIK1*-OX transgenic Arabidopsis plants that were subjected to drought stress (see below). For such, leaves were harvested at different times after the drought treatment.

For pepper plants, drought stress was imposed on six-leaf stage plants by withholding water for 10 days; for Arabidopsis, watering was withheld for 14 days on 3-week-old plants. Plants were then re-watered for 2 days, after which we calculated the survival rates (number of plants that survived). Transpirational water loss was evaluated to quantitatively determine drought tolerance. For this purpose, leaves were detached from six-leaf stage pepper and 3-week-old Arabidopsis plants and the loss of fresh weight was determined at different times.

To evaluate germination rate, primary root growth, and cotyledon greening, 100 seeds of each genotype were sown on MS agar plates containing 0 or 1 μM of ABA. We also examined ABA sensitivity on post-germination growth. For such, 3-day-old CaDIK1-OX transgenic seedlings that germinated on MS media without ABA were transferred to ABA treated media. We then measured seedling primary root growth and cotyledon greening.

### Stomatal Aperture and Leaf Temperature

We measured the size of stomatal pores and leaf temperatures according to previous study (Lim and Lee, 2016). Pepper and Arabidopsis leaf peels were placed in a stomatal opening solution (SOS: 50 mM KCl, 10 mM MES-KOH, 10 μM CaCl_2_, pH 6.15) under light. After 3 h, leaf peels were transferred to a fresh SOS containing either 0, 10, or 20 μM of ABA and incubated for another 2.5 h. For all genotypes, stomatal apertures of 100 stomata per treatment was then measured under a Nikon Eclipse 80i microscope.

To estimate leaf temperature, we used 4-week-old pepper plants with fully expanded 1st and 2nd leaves and 3-week-old Arabidopsis plants. Plants were treated with 100 μM ABA, after which thermal images were obtained using an infrared camera (T420; FLIR systems) and leaf temperatures were measured using the FLIR Tools+ ver 5.2 software.

### Quantitative Real-Time Transcription-Polymerase Chain Reaction

Pepper and Arabidopsis cDNAs were synthesized using a Transcript First Strand cDNA Synthesis kit (Roche). For the qRT-PCR analysis, we used the CFX96 Touch™ Real-Time PCR detection system (Bio-Rad) and specific primers (**Supplementary Table 1**). The PCR was programmed as follows: 95°C for 5 min, 45 cycles at 95°C for 20 s and 60°C for 20 s, and 72°C for 20 s. To calculate the relative expression levels, we used the δδCt method (Livak and Schmittgen, 2001). Arabidopsis *AtACT8* and pepper *CaACT1* were used as internal controls for normalization.

### Subcellular Localization

To determine the subcellular location of CaDIK1, the full-length coding region of *CaDIK1* without the stop codon was inserted into the GFP-fused binary vector p326GFP. A GV3101 strain of *A. tumefaciens* containing the GFP-tagged *CaDIK1* construct was combined with strain p19 (1:1 ratio; OD 600 = 0.5) and co-inﬁltrated into fully expanded leaves of 5-week-old *N. benthamiana* plants. After 2 days, the GFP signals were observed under a confocal microscope (510 UV/Vis Meta; Zeiss) equipped with the LSM Image Browser software.

## Data Availability Statement

All datasets generated for this study are included in the article/**Supplementary Material**.

## Author Contributions

JL and CL performed the experiments and analyzed the results. SL designed the experiments and wrote the manuscript. All authors contributed to the article and approved the submitted version.

## Funding

This work was supported by a grant from “the Next-Generation BioGreen 21 Program for Agriculture & Technology Development (Project No. PJ01316801)” Rural Development Administration and by the National Research Foundation of Korea (NRF) grant funded by the Korea Government (MSIT) (No. 2018R1A5A1023599, SRC), Republic of Korea.

## Conflict of Interest

The authors declare that the research was conducted in the absence of any commercial or financial relationships that could be construed as a potential conflict of interest.
