# Supplementary figures and images for: Pepper Novel Serine-Threonine Kinase CaDIK1 Regulates Drought Tolerance via Modulating ABA Sensitivity
Source: Front Plant Sci. 2020 Jul 23;11:1133. doi: 10.3389/fpls.2020.01133 (PMC7390950; doi:10.3389/fpls.2020.01133)

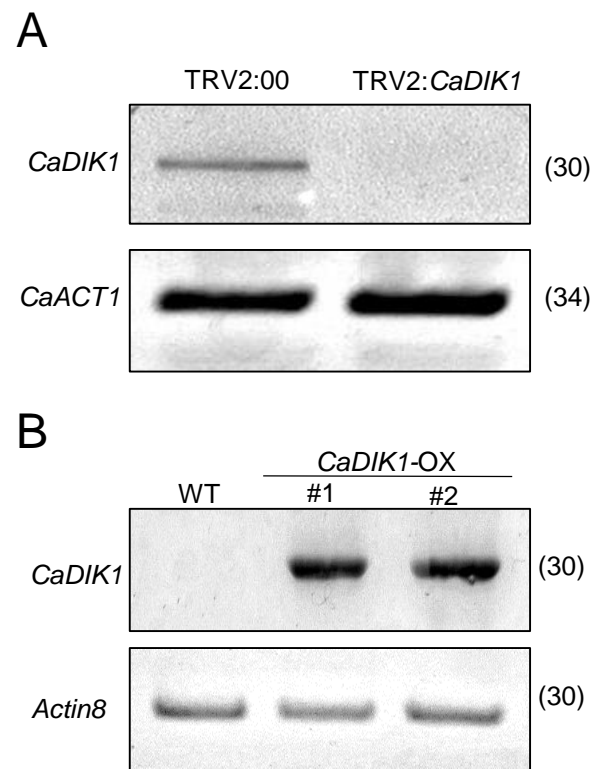

Supplementary Fig. 1 Lim et al.

Supplement: Supplementary file 1 [file Image_1.pdf]

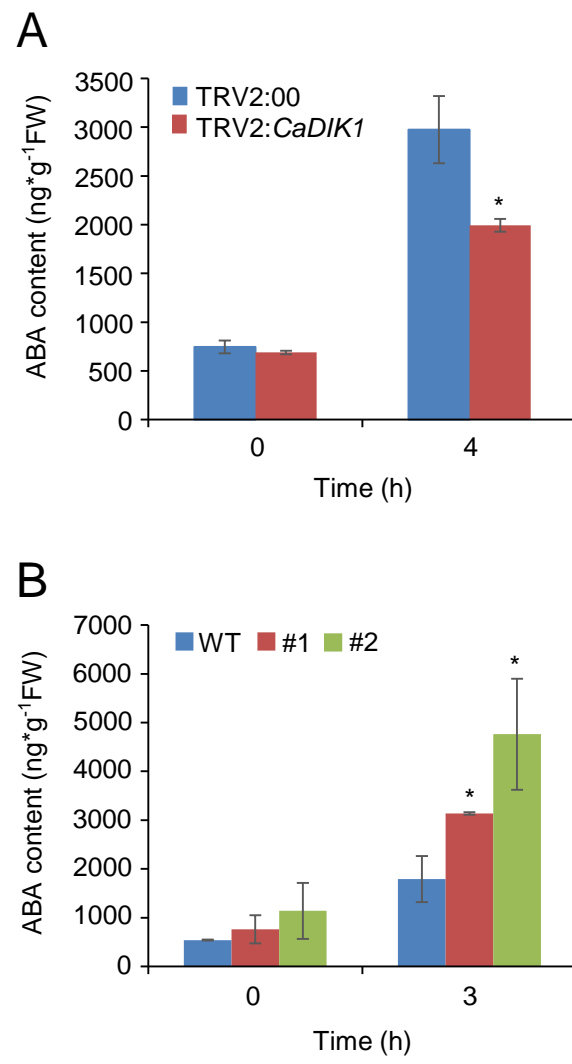

Supplementary Fig. 2 Lim et al.

Supplement: Supplementary file 2 [file Image_2.pdf]

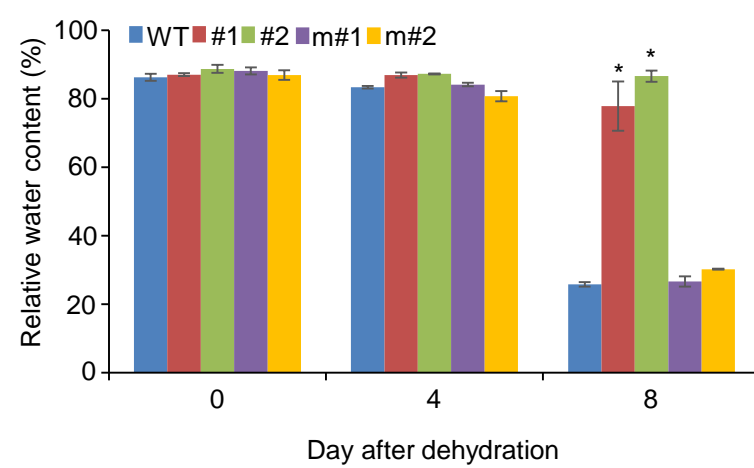

Supplementary Fig. 3 Lim et al.

Supplement: Supplementary file 3 [file Image_3.pdf]

**A**

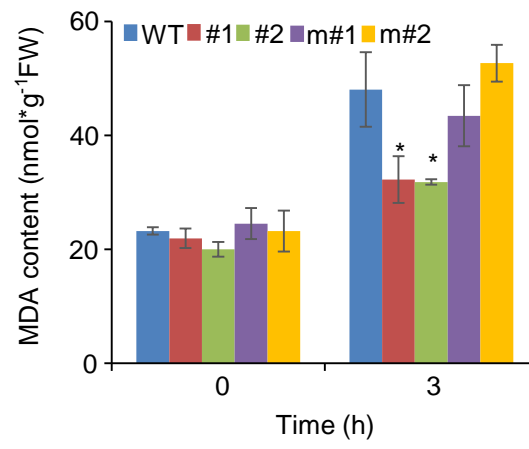

**B**

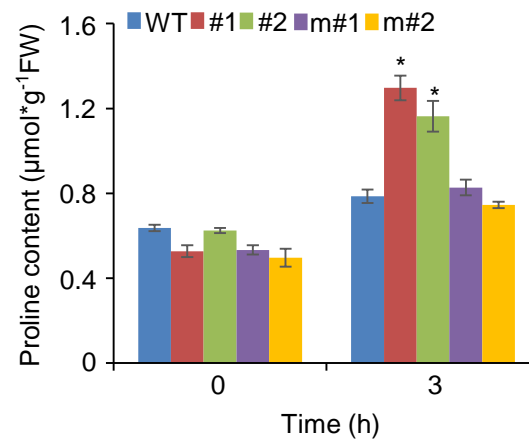

Supplementary Fig. 4 Lim et al.

Supplement: Supplementary file 4 [file Image_4.pdf]
