# Supplementary material for: Pepper Novel Serine-Threonine Kinase CaDIK1 Regulates Drought Tolerance via Modulating ABA Sensitivity
Source: Front Plant Sci. 2020 Jul 23;11:1133. doi: 10.3389/fpls.2020.01133 (PMC7390950; doi:10.3389/fpls.2020.01133)
Supplement: Supplementary file 5 [file Table_1.pdf]

Supplementary Table S1. Sequences of primers used in this study

Primer name	Primer sequence (5'-3')
For cloning	
CaDIK1	Forward: ATGGATTTTGCTTATCCTTTTGTTTGGTT
(CA11g00570[1])	Reverse: CTACTTTGTACTCGTGGTACTAGAGC
w/o stop codon	Reverse: CTTTGTACTCGTGGTACTAGAGCAAAT
Dead kinase	Forward: ATGGAAGAGAGGTTGCTGTAAACCGCCTTTACGAG
	Reverse: CTCGTAAAGGCGGTTTACAGCAACCTCTCTTCCAT
For RT-PCR	
CaDIK1	Forward: TTCTTCAAATGCAACATTACGACTA
	Reverse: GTATACTCCTGGGTAATAGGGCAAT
CaACT1	Forward: GACGTGACCTAACTGATAACCTGAT
(CA12g08730)	Reverse: CTCTCAGCACCAATGGTAATAACTT
AtActin8	Forward: CAACTATGTTCTCAGGTATTGCAGA
(At1g49240)	Reverse: GTCATGGAAACGATGTCTCTTTAGT
NCED3	Forward: ACATGGAAATCGGAGTTACAGATAG
(At3g14440)	Reverse: AGAAACAACAAACAAGAAACAGAGC
DREB2A	Forward: CTACAAAGCCTCAACTACGGAATAC
(At5g05410)	Reverse: AAACTCGGATAGAGAATCAACAGTC
RAB18	Forward: GGAAGAAGGGAATAACACAAAAGAT
(At5g66400)	Reverse: GCGTTACAAACCCTCATTATTTTTA
RD29A	Forward: CACAATCACTTGGCTCCACTGTTG
(At5g52310)	Reverse: ACCTAGTAGCTGGTATGGAGGAACT
RD29B	Forward: GTTGAAGAGTCTCCACAATCACTTG
(At5g52300)	Reverse: ATACAAATCCCCAAACTGAATAACA
ABI1	Forward: GTTTGGGATGTAATGACGGATG
(At4g26080)	Reverse: TGAACTGAGGCAGAGAGGGTCC
ABI2	Forward: AGAAAAGAGGAGAAGGAAAAGATCC
(At5g57050)	Reverse: TAAAGAGAATTTTTACCCACCATCA
HAB1	Forward: GACTACCTCTCAATGCTTGCTCTAC
(At1g72770)	Reverse: AAAAACCTGTCGAAATTAGATCCTT
For VIGS	
XbaI-CaDIK1	Forward: TCTAGATGTCTTAACACACAACCTGACTGT
XhoI-CaDIK1	Reverse: CTCGAGCTGAGCAATGTTCGGG

[1] Gene locus is from *Capsicum annuum* cv CM334 genome (1; release 1.55).
